# Association between serum ANGPLT8 levels and atherogenic index of plasma in Chinese patients with type 2 diabetes mellitus

**DOI:** 10.3389/fcvm.2026.1762073

**Published:** 2026-03-24

**Authors:** Xiaoya Sun

**Affiliations:** Department of Geriatrics, Beijing Tongren Hospital, Capital Medical University, Beijing, China

**Keywords:** ANGPTL8, atherogenic index of plasma, atherosclerosis, lipid metabolism, type 2 diabetes

## Abstract

**Aims:**

Angiopoietin-like protein 8 (ANGPTL8), a secreted adipokine, has been reported to be associated with lipid and glucose metabolism. This study aimed to evaluate the association between circulating levels of ANGPTL8 and atherogenic risk, assessed by the atherogenic index of plasma (AIP), in diabetic individuals.

**Methods:**

A total of 228 patients with type 2 diabetes (T2D) were included in this cross-sectional study. The AIP was calculated as the logarithm of the ratio of triglycerides (TG) to high-density lipoprotein cholesterol (HDL-c) and divided into two subgroups of low and high risk of CVD, as <0.11 and ≥0.11, respectively. Data including clinical characteristics and laboratory tests were collected. Serum levels of ANGPTL8 were assessed by ELISA analysis.

**Results:**

Serum concentrations of ANGPTL8 were significantly higher in the high AIP group compared with the low AIP group [293.84 (255.63–332.05) ng/mL vs. 223.05 (202.85–243.25) ng/mL; *P* < 0.001]. Serum ANGPTL8 levels were positively correlated with AIP values in patients with T2D as shown by Spearman correlation analysis (r = 0.133, *P* < 0.05). After adjusting for confounding factors, elevated ANGPTL8 levels remained significantly associated with high AIP (OR, 0.010; 95% CI: 0.994–0.999; *P* < 0.010). The optimal cutoff value of ANGPTL8 for identifying high AIP was 227.18 ng/mL (sensitivity = 68.40%; specificity = 68.00%; *P* < 0.001; AUC = 0.780; 95% CI: 0.714–0.847).

**Conclusions:**

The serum ANGPTL8 levels are elevated in T2D patients with high AIP. Increased ANGPTL8 levels are closely associated with higher AIP values, indicating a predictive value of ANGPTL8 in atherosclerosis in patients with T2D.

## 1 Introduction

Angiopoietin-like protein 8 (ANGPTL8), a secreted protein, is a member of the ANGPTL family proteins (ANGPTL1-8). ANGPTL8 is distinctive of the ANGPTLs since this protein lacks the fibrinogen-like domain (1). These structural features of ANGPTL8 suggest that its function may be different from other angiopoietin-like proteins. ANGPTL8 is predominantly expressed in the liver and adipose tissue (2, 3). The main function of ANGPTL8 is to regulate lipid metabolism through interacting with ANGPTL3, thereby inhibiting the activity of lipoprotein lipase (LPL) and further increasing triglyceride (TG) levels (4, 5). Moreover, studies in recent years have revealed the role of ANGPTL8 in many metabolic disorders, such as obesity, hypertension, coronary artery disease and diabetes mellitus (6, 7). As shown in both clinical and animal study, higher circulating levels of ANGPTL8 were observed in the type 2 diabetes (T2D) group compared to the non-diabetic group (8–12). In addition to being related to T2D, ANGPTL8 is also involved in diabetic complications (13). Therefore, ANGPTL8 plays a critical role in the occurrence and development of T2D.

Atherogenic index of plasma (AIP) is a new lipid metabolism associated index of plasma atherogenicity and also an important indicator of atherosclerosis and cardiovascular diseases (CVDs) (14). The AIP is calculated as the log-transformed ratio of TG to high-density lipoprotein cholesterol (HDL-c) in molar concentrations (15). AIP levels in patients with T2D have been reported to be markedly higher than those in healthy controls. Moreover, AIP is correlated with lipoprotein particle size and the fractional esterification rate of HDL-c (16), both of which are closely related to insulin resistance (17, 18). Currently, there is a paucity of published data investigating the association between serum ANGPTL8 levels and AIP in patients with diabetes.

The aim of this cross-sectional observational study was to investigate whether serum ANGPTL8 levels differ between diabetic patients with low and high AIP as a parameter of atherosclerosis. We found that serum ANGPTL8 may have a predictive role in atherosclerosis with T2D patients.

## 2 Methods

### 2.1 Study participants

In this study, a total of 228 adult participants with T2D who were hospitalized patients were recruited. We extracted the data of patients from the Department of Geriatric ward in Beijing Tongren Hospital from April 2023 to December 2025. Diabetes status was diagnosed according to the American Diabetes Association Criteria from the patients' medical records (19).

The exclusion criteria were as follows: diabetes other than T2D, severe hepatic disease such as chronic viral hepatitis, renal dysfunction (defined as eGFR <30 mL/min/1.73 m^2^), cancer, acute diabetic complications such as diabetic ketoacidosis, current treatment with systemic corticosteroids, and history of CVDs (including heart failure, unstable angina, myocardial infarction, stroke).

The study was conducted according to the principles of the Declaration of Helsinki and approved by the Ethics Committee of Beijing Tongren Hospital of Capital Medical University (approval No. TREC2025-KY230).

### 2.2 Data collection

All patients' age, gender, current smoking, alcohol consumption, history of hypertension, duration of diabetes mellitus, as well as their medication usage, were obtained from medical records. Drinking history was defined as drinking ≥1 time per month for a year. Body mass index (BMI) was calculated as weight [kg] divided by height squared [m^2^]. Blood pressure at the brachial artery was measured at least 3 times and the mean value of the 3 measurements was calculated. Arterial hypertension was defined according to the current guidelines if systolic blood pressure (SBP) was ≥140 mmHg and/or diastolic blood pressure (DBP) was ≥90 mmHg after at least 0.5 h of rest and/or if patients were on antihypertensive treatment. The calculation of the homeostatic model assessment for insulin sensitivity (IS_HOMA−CP_) was as follows: IS_HOMA−CP_ = 22.5/ (glucose×C-peptide in fasting status).

All the laboratory assessments, except plasma ANGPTL8 levels, were conducted in the central laboratory using standard methods. Biochemical tests included fasting blood glucose (FBG), fasting C-peptide (FCP), 2-h postprandial blood glucose (2hPG), glycosylated hemoglobin A1c (HbA1C), UA (uric acid), total cholesterol (TC), TG, HDL-c, low density lipoprotein cholesterol (LDL-c), lipoprotein a (LPa).

### 2.3 Quantification of ANGPTL8

Samples were collected in EDTA-containing tubes after an overnight fast. Plasma was centrifuged for 15 min at 3,000 rpm at 4℃ and stored at −80℃. Serum ANGPTL8 levels were measured using enzyme-linked immunosorbent assay (ELISA) kit (Ruixin Biotechnology, Quanzhou, China) according to the manufacturer's instruction. Absorbance measurements were assayed in a microplate reader at 450 nm and the concentrations were calculated using a standard curve.

### 2.4 Calculation of AIP

AIP was defined using the formula of Log TG to HDL logarithm *Log* $\left(\frac{TG}{HDL-c}\right)$ (20). According to the AIP cutoff values (0.11), diabetic patients were divided into high AIP group (*n* = 92) and low AIP group (*n* = 136).

### 2.5 Statistical analysis

All data were processed using Statistical Package for the Social Sciences (IBM SPSS software version 22.0 for windows, Armoonk, New York, USA) and Graphpad Prism software 8.0 (San Diego, CA, USA). *P*-values < 0.05 (two-tailed) were considered statistically significant.

The Shapiro–Wilk test was used to examine the normal distribution of the continuous variables. Continuous variables are presented as means ± standard deviation (SD) and categorial variables are presented as median with interquartile ranges (25th and 75th percentiles). Student's *t*-test was used to compare continuous data with normal distribution. Mann–Whitney *U*-test was to compare non-normally distributed variables. Categorial variables were expressed as the number and percentage of cases and Chi-squared (*χ*^2^) tests were used to compare data difference between groups. Spearman correlation analysis was performed to explore the relationship between AIP and the ANGPTL8 levels. Univariable logistic regression analysis was used to select all the variables between the high AIP and low AIP groups. Subsequently, any variables that were verified to be significant (*P* < 0.05) in the univariable analysis were chosen to enter into models and included in the multivariate stepwise binary logistic regression analysis to verify the combined effect of these variables on the odds ratios (OR) and 95% confidence intervals (CI) of ANGPTL8. The receiver operating characteristic (ROC) curve was used to identify the ability of ANGPTL8 to predict atherosclerosis in patients with T2D.

## 3 Results

### 3.1 Baseline characteristics between the high AIP group and the low AIP group

The baseline characteristics of the two groups were presented in Table 1. A total of 228 patients with T2D (92 patients with high AIP and 136 patients with low AIP) were enrolled in this study. The mean age of the high AIP group was 69.21 years and the proportion of male sex was 68.48%, whereas the mean age of the low AIP group was 72.36 years and the percentage of male sex was 66.18%. No significant differences were found in gender, diabetes duration, BMI, SBP, DBP, antidiabetic treatment, history of hypertension, the use of statins, current smoking and history of drinking between the high AIP and the low AIP group. The levels of FBG, FCP, 2hPG, HbA1c, IS_HOMA−cp_ and LPa were similar between the two groups. Diabetic patients with high AIP had older age, relatively lower HDL-c levels and higher levels of UA, TC, TG, LDL-c compared with low AIP patients.

**Table 1:** Baseline characteristics between type 2 diabetic patients with high AIP and those with low AIP.

Variables	High AIP (*n* *=* 92)	Low AIP (*n* *=* 136)	*P*-value
Age (years)	69.21 ± 10.04	72.36 ± 10.29	0.023*
Men, *n* (%)	63 (68.48)	90 (66.18)	0.717
Diabetes duration (months)	13.53 (11.35–15.71)	13.37 (11.65–15.09)	0.959
BMI (kg/m^2^)	24.79 (24.20–25.37)	24.41 (23.77–25.04)	0.384
SBP (mmHg)	131.97 ± 17.49	130.95 ± 16.53	0.657
DBP (mmHg)	74.75 ± 10.46	72.6 ± 10.38	0.118
Antidiabetic treatment			
Lifestyleinterventionalone, *n* (%)	21 (22.83)	36 (26.47)	0.533
Insulin treatments, *n* (%)	31 (33.70)	38 (27.94)	0.353
Insulin-secretagogues, *n* (%)	10 (10.87)	13 (9.56)	0.747
Insulin-sensitisers, *n* (%)	60 (65.22)	72 (52.94)	0.065
SGLT2 inhibitors, *n* (%)	22 (23.91)	21 (15.44)	0.109
Hypertention, *n* (%)	70 (76.09)	109 (80.15)	0.399
Statins, *n* (%)	58 (63.0)	92 (67.65)	0.472
Current smoking, *n* (%)	22 (23.91)	28 (20.59)	0.552
Drinking, *n* (%)	19 (20.65)	21 (15.44)	0.310
FBG (mmol/L)	8.59 (6.32–10.85)	7.25 (6.81–7.68)	0.087
FCP (ng/mL)	2.66 (2.32–3.01)	2.41 (2.00–2.83)	0.371
2hPG (mmol/L)	11.60 (10.78–12.41)	11.24 (10.54–11.94)	0.302
HbA1c (%)	8.01 (7.57–8.45)	7.79 (7.46–8.11)	0.332
IS_HOMA−cp_	4.31 (3.23–5.40)	5.56 (3.56–7.57)	0.664
UA (*μ*mol/L)	367.10 (348.35–385.85)	338.74 (325.44–352.05)	0.018*
TC (mmol/L)	4.34 (4.08–4.60)	3.79 (3.62–3.97)	0.000***
TG (mmol/L)	2.41 (1.95–2.86)	0.97 (0.92–1.02)	0.000***
HDL-c (mmol/L)	1.00 (0.96–1.05)	1.29 (1.24–1.34)	0.000***
LDL-c (mmol/L)	2.50 (2.31–2.70)	2.11 (1.97–2.26)	0.001**
LPa (mg/L)	21.40 (16.29–26.51)	21.05 (17.08–25.02)	0.635
ANGPTL8 (ng/mL)	293.84 (255.63–332.05)	223.05 (202.85–243.25)	0.001**
lnAIP	0.33 ± 0.20	−0.14 ± 0.19	0.000***

Values are shown as mea*n* ± standard deviation (SD), median (25th, 75th) or numbers (percentages).

BMI, body mass index; SBP, systolic blood pressure; DBP, diastolic blood pressure; SGLT2 inhibitors, sodium-glucose cotransporter 2 inhibitors; FBG, fasting blood glucose; FCP, fasting C-peptide; 2hPG, 2-h postprandial blood glucose; HbA1c, glycosylated hemoglobin A1c; IS_HOMA−CP_, homeostasis model assessment for insulin sensitivity calculated using glucose and C-peptide in fasting status; UA, uric acid; TC, total cholesterol; TG, triglycerides; HDL-c, high density lipoprotein cholesterol; LDL-c, low density lipoprotein cholesterol; LPa, lipoprotein a; ANGPTL8, angiopoietin-like protein 8; AIP, atherogenic index of plasma. A two-tailed *p* < 0.05 was considered statistically significant.

* *p* < 0.05,

** *p* < 0.01,

*** *p* < 0.001.

### 3.2 Serum ANGPTL8 levels in patients with T2D and its correlation with AIP

**Figure 1:**
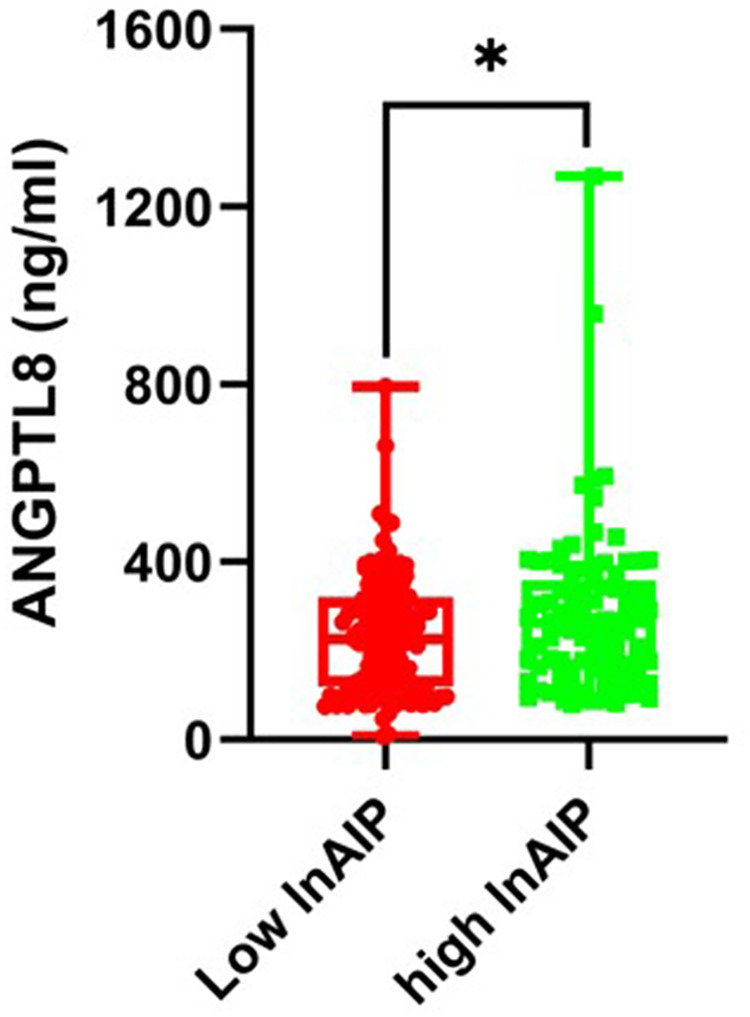
Analysis of plasma ANGPTL8 levels on diabetic patients with low lnAIP or those with high lnAIP. Box-and-whisker plot presents all points as well as minimum, maximum, median and quartiles. ANGPTL8, angiopoietin-like protein 8; AIP, atherogenic index of plasma. * *p* < 0.05.

**Figure 2:**
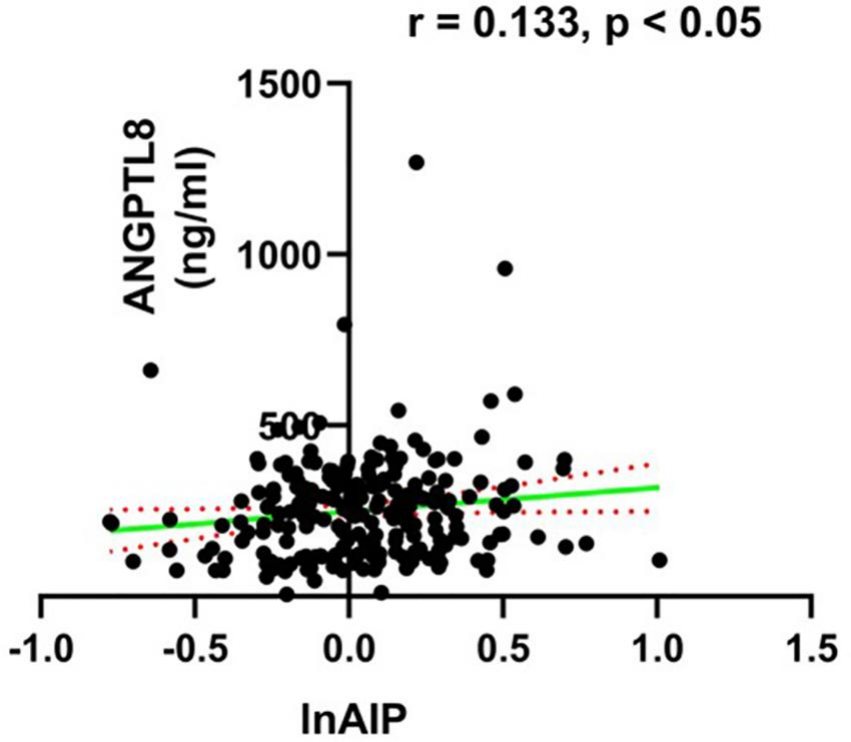
The relationship between ANGPTL8 and lnAIP in diabetic patients. ANGPTL8, angiopoietin-like protein 8; AIP, atherogenic index of plasma.

Serum concentrations of ANGPTL8 in diabetic patients with high AIP were significantly higher in comparison to those with low AIP [293.84 (255.63–332.05) ng/mL vs. 223.05 (202.85–243.25) ng/mL; *P* = 0.001; Figure 1]. Correlation analysis revealed that serum ANGPTL8 levels were highly correlated with AIP in diabetic individuals (Spearman's *r* = 0.133, *P* *<* 0.05, Figure 2).

### 3.3 Serum levels of ANGPTL8 were positively related to AIP in patients with T2D

As showed in Table 2, univariate logistic regression analysis demonstrated that higher levels of ANGPTL8 were notably associated with high AIP in T2D patients (OR, 0.996; 95% CI 0.004–0.999; *P* = 0.001). After controlling for age, sex, BMI, smoking, diabetic duration, SBP, DBP, hypertension, statins, UA, UC, LDL, LPa and anti-diabetic treatment in models 2, 3 and 4, a positive association between the serum ANGPTL8 concentration and AIP in diabetic individuals remained (OR, 0.010; 95% CI 0.004–0.999; *P* = 0.010). As a result, the serum ANGPTL8 levels were independently and positively associated with AIP in patients with T2D.

**Table 2:** Odds ratios of plasma ANGPTL8 levels for type 2 diabetic individuals with high AIP or low AIP.

Models	OR	95% Confidence interval	*p*-value
Model 1: unadjusted	0.996	0.994–0.999	0.001^**^
Model 2: age, sex, BMI, smoking and diabetic duration	0.997	0.994–0.999	0.004^**^
Model 3: Model 2 + SBP, DBP, hypertension, statins, UA, TC, LDL and LPa	0.997	0.994–0.999	0.009^**^
Model 4: Model 3 + antidiabetic treatments	0.01	0.994–0.999	0.010^*^

Model 1 was unadjusted;.

Model 2 was adjusted for age, sex, BMI, smoking, diabetic duration;.

Model 3 was adjusted for age, sex, BMI, smoking, diabetic duration, SBP, DBP, hypertension, statins, UA, TC, LDL-c and LPa;.

Model 4 was adjusted for age, sex, BMI, smoking, diabetic duration, SBP, DBP, hypertension, statins, UA, TC, LDL-c, LPa, antidiabetic treatment.

ANGPTL8, angiopoietin-like protein 8; AIP, atherogenic index of plasma; BMI, body mass index; SBP, systolic blood pressure; DBP, diastolic blood pressure; UA, uric acid; TC, total cholesterol; LDL-c, low density lipoprotein cholesterol; LPa, lipoprotein a.

* *p* < 0.05,

** *p* < 0.01,

### 3.4 Diagnostic performance of ANGPTL8 as a predictor of AIP in patients with diabetes

**Figure 3:**
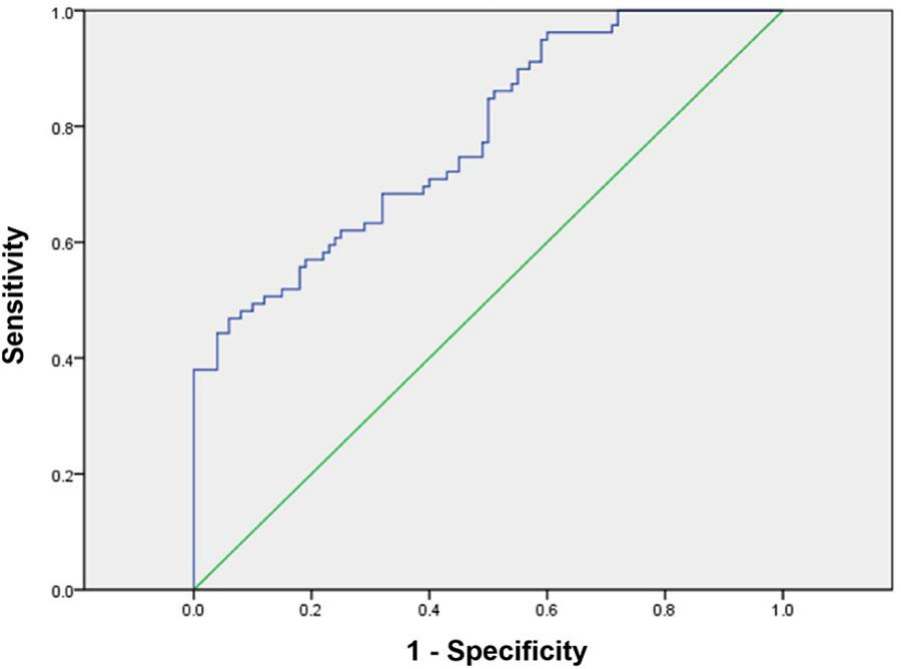
ROC curve for ANGPTL8 in predicting arterial stiffness measured by AIP in diabetic patients. (AUC, 0.780; 95% CI, 0.714–0.847; *p* < 0.001). The best cutoff value was 227.18 ng/mL, with the best sensitivity (68.4%) and specificity (68.0%). ANGPTL8, angiopoietin-like protein 8; AIP, atherogenic index of plasma; ROC, receiver operating characteristic; AUC, the area under the curve; CI, confidence interval.

Figure 3 illustrates the ROC curve analysis for the prediction of AIP in T2D. ANGPTL8 level >227.18 ng/mL was the optimal cutoff value for accurate prediction of AIP >0.11 with a sensitivity of 68.4%, specificity of 68.0%. The corresponding area under the curve value was 0.780 (AUC, 0.780; 95% CI, 0.714–0.847; *P* < 0.001).

## 4 Discussion

Our study revealed that there is an independent positive correlation between plasma ANGPTL8 levels and high AIP in diabetic patients. Furthermore, higher plasma ANGPTL8 concentrations could be used to predict atherosclerosis in diabetic individuals, indicating a crucial role for ANGPTL8 in the atherosclerosis process in the diabetic population.

Recently, many investigations demonstrated that ANGPTL8 performed vital roles in cardio-metabolic diseases, such as obesity, hypertension, and diabetes mellitus. In a large cohort study, Abu-Farha et al. found that the ANGPTL8 was approximately 3-fold higher in type 2 diabetics than non-diabetics and patients in the highest tertiles of ANGPTL8 showed approximately 6 times higher risk of developing T2D, indicating that plasma ANGPTL8 could be considered as an independent predictor of T2D (8). In rodent studies, db/db diabetic mouse models showed higher levels of ANGPTL8 compared to those in control mice (21). These results are consistent with the findings of our study. However, Gomez-Ambrosis et al. showed that the serum ANGPTL8 concentrations were significantly increased in overweight but not in diabetic and normal glycemic individuals (22). Another clinical trial reported no significant difference regarding ANGPTL8 in diabetics vs. non-diabetics (23). These discrepancies are probably due to the sample size and analytical formats.

AIP, a lipid marker, was initially constructed as a novel and reliable predictor of plasma atherosclerosis to predict the risk of CVDs (14). In recent years, numerous studies have revealed that the association between the AIP and the risk of insulin resistance-related metabolic diseases, such as obesity, metabolic syndrome, prediabetes and diabetes (24, 25). Clinical trials from different countries including Chinese, Korean and Iranian populations found that higher AIP could be an important predictor of the incidence of insulin resistance and T2D (26–28). Hence, we divided the diabetic participants into 2 groups with different levels of AIP. In the current study, we corroborated that the serum ANGPTL8 concentrations were higher in individuals with high AIP than those with low AIP and were positively correlated with the AIP values, implying that ANGPTL8 might become a new biomarker for early atherosclerosis in T2D patients.

Although it is unknown how exactly increased ANGPTL8 contributes to atherosclerosis in the diabetic subjects, the following ones can be suggested. Intercellular adhesion molecule 1 (ICAM-1), a marker of endothelial dysfunction, is an intercellular adhesion molecule predominantly expressed in endothelial cells that promotes a proinflammatory state. Reza Fadaei et al. revealed that there was an independent association between ANGPTL8 levels and ICAM expression (29). Moreover, inflammation cytokines, such as tumor necrosis factor alpha (TNF-α) and high sensitivity C-reactive protein (hsCRP) had been verified to have significant positive correlations with ANGPTL expression (29–32). Collectively, it might imply that ANGPTL8 might be involved and play a certain role in the inflammatory process. In addition, it is widely recognized that chronic inflammation is also a pivotal contributor to the pathogenesis and development of atherosclerosis and T2D (33–37). In our study, we observed a substantially increased ANGPTL8 levels of high AIP than in the low AIP group in T2D patients, even after accounting for the variables that maybe relevant. Hence, we speculated that inflammation might be the underlying mechanism involved in ANGPTL8 and atherosclerosis and further mechanistic research is needed.

This study has several inevitable limitations that need to be considered. First, because this study was a cross-sectional design, even though we have multiple models for evaluation, we are still unable to determine the causal relationships between the elevated serum ANGPTL8 levels and increased risk of atherosclerosis as assessed by AIP. Second, the sample size of the present study was relatively small, so there is a possibility that the statistical power and strength of the evidence were reduced. Third, our research was a single-centre study conducted only among Chinese participants, thus it is uncertain that the results of this study can be generalized to non-Chinese populations, especially in non-Asian populations. Lastly, the influence of antidiabetic treatment on AIP values is unknown. Therefore, further large-scale prospective studies need to be conducted to better investigate the correlation between ANGPTL8 levels and the risk of atherosclerosis in T2D patients.

## 5 Conclusions

In summary, this study demonstrated that serum levels of ANGPTL8 are elevated in diabetic patients with high AIP. More importantly, ANGPTL8 levels were indenpendently and positively associated with AIP, indicating that plasma ANGPTL8 may have a predictive role in atherosclerosis in patients with T2D. Targeting ANGPTL8 is a promising therapeutic strategy for atherosclerosis during T2D.

## Data Availability

The datasets presented in this article are not readily available because anyone can apply to the database through sending email to the first author. Requests to access the datasets should be directed to sunxiaoya2021@163.com.
